# Incredible use of plant-derived bioactives as anticancer agents

**DOI:** 10.1039/d4ra05089d

**Published:** 2025-01-20

**Authors:** Kiran Kangra, Saloni Kakkar, Vineet Mittal, Virender Kumar, Navidha Aggarwal, Hitesh Chopra, Tabarak Malik, Vandana Garg

**Affiliations:** a Department of Pharmaceutical Sciences, Maharshi Dayanand University Rohtak 124001 India kirankangra90@gmail.com drsaloni.pharma@mdurohtak.ac.in drvineet.pharma@mdurohtak.ac.in drvandana.pharma@mdurohtak.ac.in; b College of Pharmacy, Pandit Bhagwat Dayal Sharma University of Health Sciences Rohtak 124001 India sachdeva.virender5@gmail.com; c MM College of Pharmacy, Maharishi Markandeshwar (Deemed to be University) Mullana Ambala 133207 Haryana India; d Department of Biosciences, Saveetha School of Engineering, Saveetha Institute of Medical and Technical Sciences Chennai 602105 Tamil Nadu India; e Department of Biomedical Sciences, Jimma University Jimma Ethiopia tabarak.malik@ju.edu.et; f Division of Research & Development, Lovely Professional University Phagwara Punjab-144411 India

## Abstract

Cancer is a major global concern. Despite considerable advancements in cancer therapy and control, there are still large gaps and requirements for development. In recent years, various naturally occurring anticancer drugs have been derived from natural resources, such as alkaloids, glycosides, terpenes, terpenoids, flavones, and polyphenols. Plant-derived substances exhibit their anticancer potential through antiproliferative activity, cytotoxicity, apoptosis, angiogenesis and cell cycle arrest. Natural compounds can affect the molecular activity of cells through various signaling pathways, like the cell cycle pathway, STAT-3 pathway, PI3K/Akt, and Ras/MAP-kinase pathways. Capsaicin, ouabain, and lycopene show their anticancer potential through the STAT-3 pathway in breast, colorectal, pancreatic, lung, cervical, ovarian and colon cancers. Epigallocatechin gallate and emodin target the JNK protein in skin, breast, and lung cancers, while berberine, evodiamine, lycorine, and astragalin exhibit anticancer activity against breast, liver, prostate, pancreatic and skin cancers and leukemia through the PI3K/Akt and Ras/MAP-kinase pathways. *In vitro*/*in vivo* investigations revealed that secondary metabolites suppress cancer cells by causing DNA damage and activating apoptosis-inducing enzymes. After a meticulous literature review, the anti-cancer potential, mode of action, and clinical trials of 144 bioactive compounds and their synthetic analogues are included in the present work, which could pave the way for using plant-derived bioactives as anticancer agents.

## Abstract 2

Bioactives for the treatment of cancer.

## Graphical abstract

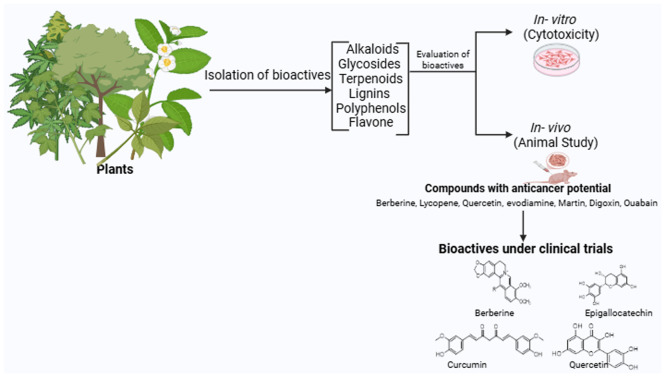

## 1 Introduction

Among the non-communicable diseases, cancer is the second most life-threatening disease after cardiovascular diseases.^1^ It is caused by a combination of genetic factors, environmental stress on cellular activity,^2^ obesity, poor diet, excessive alcohol intake, smoking, and vitamin B_12_ deficiency.^3^ According to Sung *et al.*, transitional cases (64%) would increase significantly more than transitioned cases (32%), reaching 28.4 million cases globally in 2040, with an increase of 47% cases from 2020. Approximately 10 million people would die from cancer in 2024, out of which approximately 19.3 million would be new cases.^4^ According to the site of occurrence, there are 131 different types of cancers, including skin cancer, lung cancer, oral cancer, and breast cancer.^5^ It is predicted that there would be 2.3 million more cases of female breast cancer (11.7%), followed by lung (11.4%), colorectal (10.1%), prostate (7.3%), and stomach (5.6%) cancers. Different types of cancers are caused by a variety of variables. Particularly, in the case of skin cancer, ozone depletion, melanin and microbial impact are responsible for its onset.^6^ Lung cancer is primarily caused by smoking but can also occur in non-smokers owing to other factors, like exposure to radon gas or secondhand smoke. Prostate cancer affects men and is one of the most common cancers in older men. Colorectal cancer affects the colon or rectum and is more common in older adults. It can be developed due to hereditary reasons or develop sporadically. Breast cancer occurs primarily in women but can also affect men. Thus, the pathophysiology of cancer involves a multitude of genetic, molecular, and environmental factors. Cancer arises from mutations in the DNA of cells that disrupt normal control mechanisms governing cell growth and division. These mutations can be inherited or acquired over time due to exposure to carcinogens such as chemicals, radiation, or viruses.

### 1.1 Pathophysiology of cancer

Cancer is a four-step process involving mutation along with cell's proliferative, survival, invasion, and metastatic capacities. In cancer, the cell's genetic system (DNA) and anti-tumor genes are suppressed by environmental factors or unhealthy diet, smoking, drinking obesity *etc.* Tumor suppressor gene inactivation is a natural physiological reaction of the organism and the cancer develops when this reaction becomes pathologic.^7^ Except for histological types, almost all cancers share basic pathogenesis. From extensive research, it is evident that the genetic system is involved in the development of malignant tumors. Due to inhibition of angiogenesis and alteration of cells.^8^

**Fig. 1:**
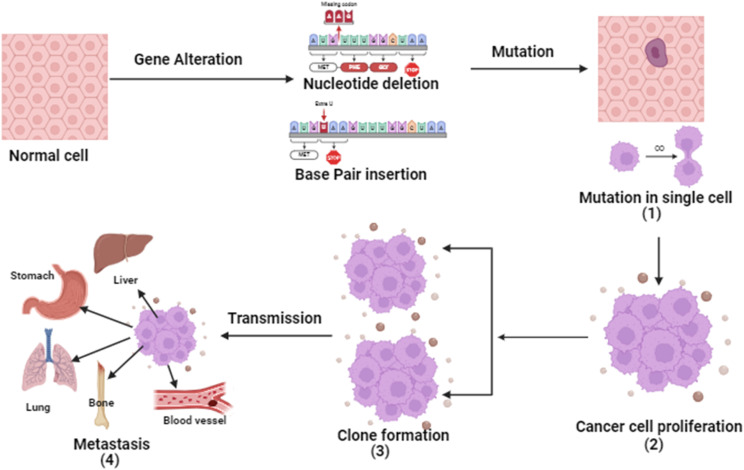
Flow-chart indicating the basic pathophysiology of different types of cancers.

The initial stage in the progression of cancer involves the occurrence of a mutation and the subsequent formation of a tumor. This process occurs when a genetic alteration triggers a mutation within a cell, leading to the growth of tumor cells. Following this, the mutation induces cell proliferation and the advancement of the tumor as the mutated cells rapidly multiply and divide, ultimately becoming dominant within the tumor cell population.^9^ Subsequently, clonal selection occurs among the proliferating cells, resulting in the generation of a new clone of rapidly growing cells with distinct characteristics. This step is repeated throughout the development of the tumor. Finally, metastasis occurs, wherein cancer cells detach from the primary tumor and travel through either the bloodstream or the lymphatic system to distant areas of the body.^10^ Consequently, these cells continue to multiply in the new locations, ultimately giving rise to new tumors composed of cells that bear resemblance to the original tissue. The propensity of tumors to metastasis is a major factor in the lethality of some malignancies, such as pancreatic and uveal cancers.^11^ The basic pathophysiology of cancer is described in Fig. 1.

## 2 Review methodology

**Fig. 2:**
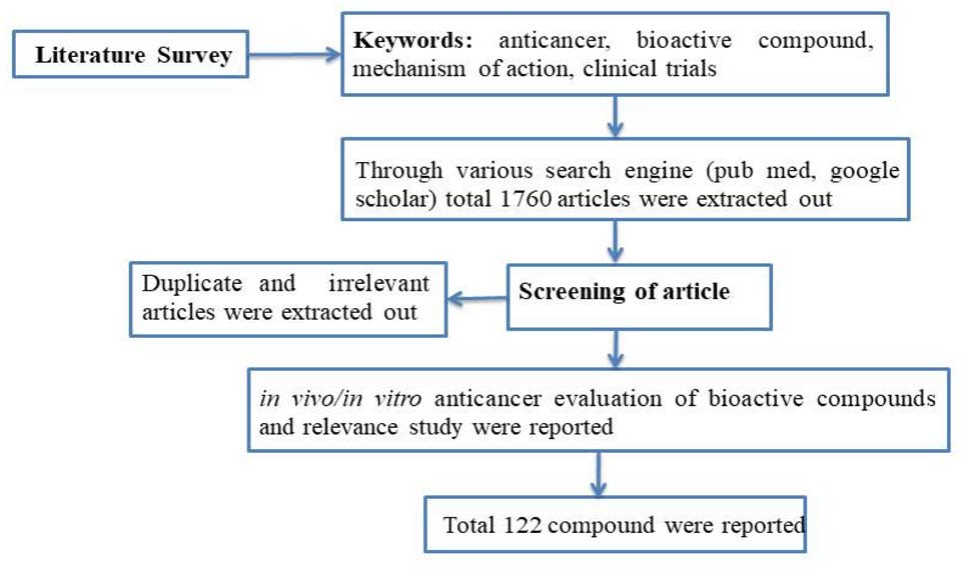
Flow chart summarizing the review methodology.

A concise summary of the methodology employed in this review is shown in Fig. 2. A comprehensive search was conducted using various search engines such as PubMed, Google Scholar, ScienceDirect, Scopus, Web of Science, and Chemical Abstracts. The search utilized different keywords including “anti-cancer”, “phytochemical”, “plant bioactive”, “clinical trials”, “mechanism of action”, and more. Irrelevant, duplicate, and incomplete data were excluded, while the literature pertaining to the *in vitro* or *in vivo* anticancer potential of plant-based bioactives was included by studying the 1600–1700 review and research article. Additionally, this review focused on articles that described the mechanism of action and clinical trial data for the anti-cancer potential of herbal compounds. The present article primarily reviews data published within the past decade.

## 3 Role of traditional plants and derived bioactives in cancer

Plants are used to cure many ailments and natural or plant-based medications are preferred by 60–70 percent of the population over synthetic medicines for one reason or the other. These plants may aid the patient resistance to sickness by arbitrating physiological homeostasis and retraining the body tissues.^12^ Based on the traditional uses of plants and scientific reports, a lot of research has also been dedicated to the study of plants in order to cure cancer, and several plants have been successfully used in the treatment of cancer.^13^ Isolated phytoconstituents from these plants such as vincristine, vinblastine, chlorogenic acid, gingerol, apigenin, catechin, gallic acid, cinnamic acid, and podophyllotoxin, along with their derivatives and analogues, are used for the treatment of cancer by inhibiting several signaling pathways. Different types of tumors have altered cell signaling pathways (cell death pathways: apoptosis and autophagy, embryonic developmental pathways: Notch, Wnt, Hedgehog, Janus kinase pathway, signal transducer and activator of the transcription factor pathway and RAF/MAPK pathway). Cells integrate the signals received from various growth factors and receptors to control different cellular functions, including cell motility, differentiation, architecture, and polarity. Signalling pathways control cellular growth and induce various alterations in various cell types.^14^

**Fig. 3:**
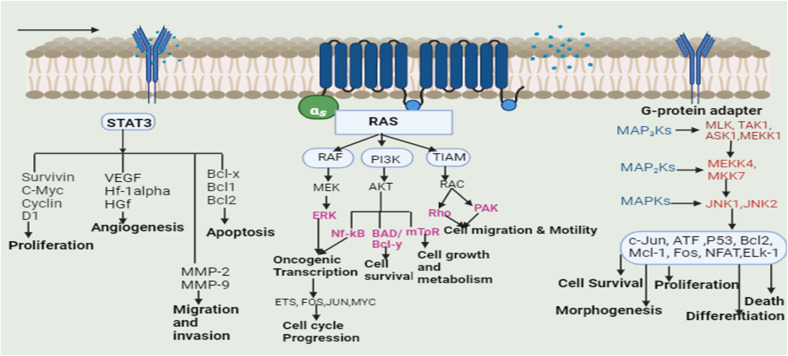
Signal transducers and activators of the transcription (STAT3) pathway, Ras/Raf/MAPK (mitogen-activated protein kinase) pathway, and JNK (c-Jun N-terminal kinases) pathway in the development of cancer/tumor [JNK (c-Jun N-terminal kinases), signal transducers and activators of transcription (STAT3), vascular endothelial growth factor (VEGF), hepatocyte nuclear factor-1 alpha (HF-1 alpha), hepatocyte growth factor (HGF), B-cell lymphoma (Bcl), matrix metalloproteinases (MMP), RAF (rapidly accelerated fibrosarcoma), MEK/MAPK (mitogen activated protein kinase), ERK (extracellular signal-regulated kinase), PI3K (phosphatidylinositol-3 kinase), Akt (Akt kinase), mechanistic target of rapamycin (mTOR), nuclear factor-κB (Nf-kB), Rho GTPases (Rho), p21-activated kinases (PAK), early tumour shrinkage (ETS), FOS protooncogene (FOS), JUN protooncogene (JUN), mixed-lineage kinase (MLK), transforming growth factor-β-activated kinase 1 (TAK1), apoptosis signal-regulating kinase 1 (ASK1), nuclear factor of activated T cells (NFAT), and ETS-like protein 1 (ELK1)].

The transcription (STAT3) pathway with signal transducers and activators is a major intrinsic pathway in cancer development (Fig. 3). It transmits intracellular signals that are normally generated at cell surface receptors to the nucleus. STAT3 activation involved a number of human tumors, including haematological and solid tumors. The evidence suggests that oncogenic cell transformation activates STAT-3, providing the survival signal. The dysfunctioning of STAT-3 during mammary gland involution demonstrates that it has proapoptotic functions. Functioning STAT-3 can prevent apoptosis in most cells. These effects are arbitrated by STAT-3-regulated cell survival gene products, *i.e.* Bcl Bcl1, Bcl-2, Survivin, Mcl-1, and cIAP2. Thus, inhibiting the STAT-3 activation can reduce the activity of these gene products, thereby increasing apoptosis.^15^ Furthermore, the master protein kinases known as c-Jun N-terminal kinases (JNKs) control a variety of physiological processes, such as inflammatory reactions, morphogenesis, cell proliferation, differentiation, survival, and death. It is becoming clear that persistent JNK activation contributes to cancer development and progression. Further, RAS proteins can interact with other well-known effectors such as phosphatidyl inositol 3-kinases (PI3Ks) *via* the RAF/MAPK pathway (PI3Ks). The interaction of different RAS proteins with PI3Ks could lead to DNA damage, and finally, to tumor development.

In the present study, different bio-actives from various categories that are reported to possess anti-cancer potential against various cell lines and in experimental animals are summarised.

**Fig. 4:**
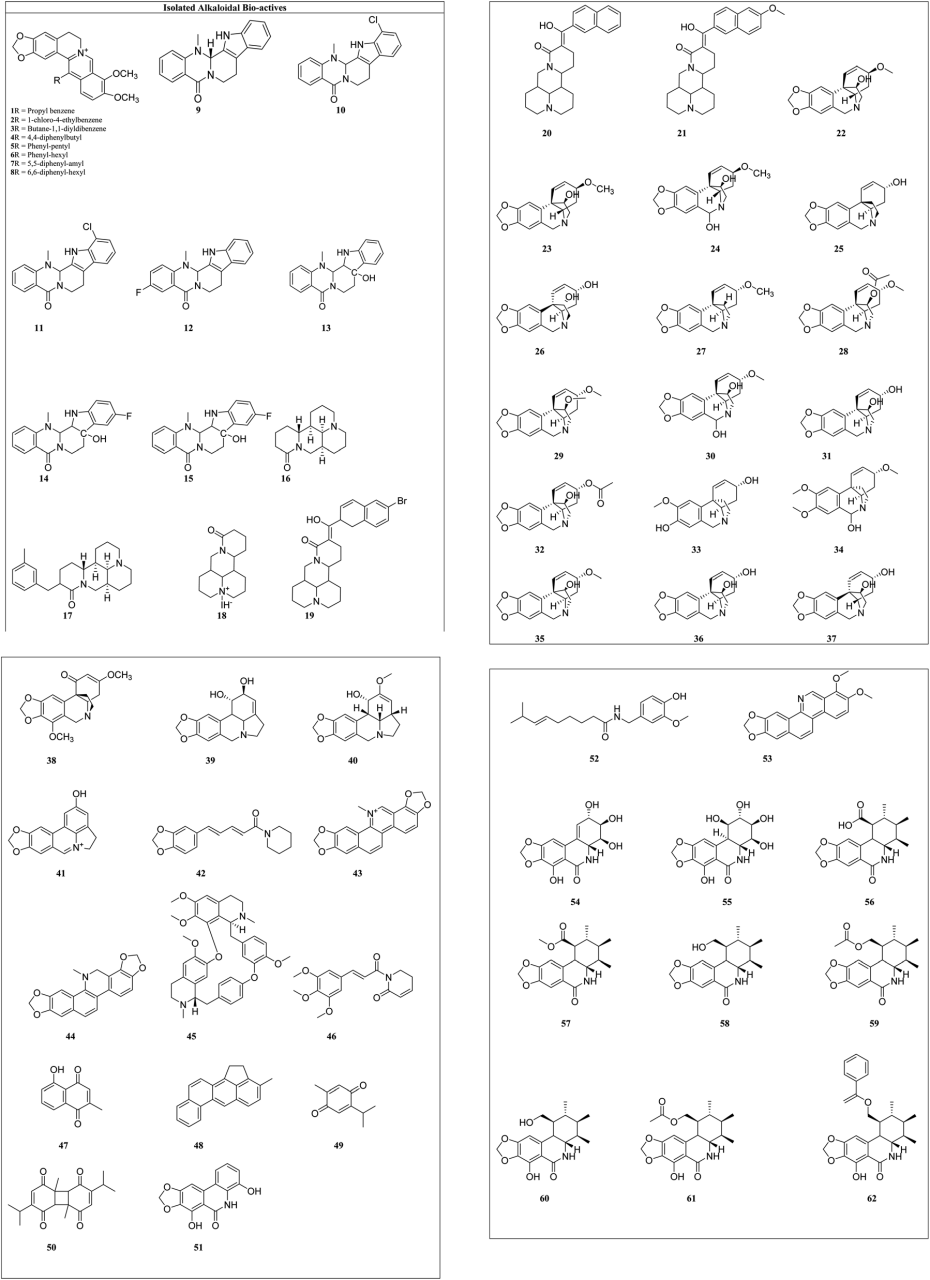
Structures of isolated alkaloids.

Alkaloids: these are the largest group of phytochemicals with a heterocyclic ring structure and at least one nitrogen atom. To distinguish various alkaloids, a categorization based on biosynthetic pathways is commonly used. Alkaloids can be found in all types of plants, although they are most prevalent in the Ranunculaceae, Leguminosae, Papaveraceae, Menispermaceae, and Loganiaceae families.^16^ Vinca alkaloids (vincristine, vinblastine, vinorelbine, vindesine, and vinflunine) were the first microtubule-targeting agents (MTAs) and approved for clinical use in hematological and lymphatic neoplasms.^17^ Various alkaloids from plant sources and their synthetic analogues with cytotoxicity on different cell lines are reported in Table 1 and the structures of isolated alkaloids are shown in Fig. 4.

**Table 1:** Various isolated alkaloids, their biological sources and reported IC_50_ against different types of cancer cell lines

Isolated compound	Alkaloid (biological source)	Cancer cell line along with IC_50_ (nM) value	References
1	*Berberis aetnensis* (Berberidaceae)	MCF-7-230	18 and 19
2		HepG2-170	
3		LNCaP-190	
4		PC-3165	
5		MHCC97-L-400	
6		MDA-MB21-250	
7		HTB-94-200	
8		SMMC-7721-180	
9	*Evodia rutaecarpa* (Rutaceae)	MDA-MB-435-49	20
10		HCT116-90	
11		U20S-26	
12		Panc-1-39	
13		PC-3-65	
14		HL-60-60	
15		Saos-2-95	
16	*Sophora flavescens* (Fabaceae)	A549-200	21 and 22
17		HepG2-250	
18		Panc-1-170	
19		CCRFCEM-250	
20		SGC7901-209	
21		PC-3-170, DU145-175	
22	*Galanthus nivalis* (Amaryllidaceae)	A-431-90	23 and 24
23		A549-255	
24		BCA-1-200	
25		B16F10-200	
26		CEM-140	
27		HT29-230	
28		HeLa-120	
29		HepG2-156	
30		Hs683-130	
31		HL-60-145	
32		B16F10-250	
33		CEM-180	
34		BCA-1-220	
35		A549-275	
36		HT29-280	
37	*Crinum bulbispermum* (Amaryllidaceae)	U373-280	25 and 26
		HL-60-120	
38	*Boophone disticha* (Amaryllidaceae)	HeLa-150	27
		G-361-250	
		MCF-7-200	
		K562-280	
39	*Hymenocallis littoralis* (Amaryllidaceae)	HL-60-150	28–30
		K562-180	
		PC-3M-200	
40	*Amaryllis belladonna* L., (Amaryllidaceae)	A549-280	
		OE21-220	
		B16F10-290	
		U373-360	
41	*Nerine bowdenii* (Amaryllidaceae)	HL-60-200	30
		U937-290	
		K562-360	
		MOLT-4-270	
		LXFL 529L-240	
42	*Piper nigrum* L. (Piperaceae)	DU145-150	31 and 32
		HT-29-180, Caco-2-200, SW480-220	
		HRT-18-220	
		A549-140	
43	*Sanguinaria canadensis* (Papaveraceae)	DU145-210	33
		BEL-7402-280	
44		Hela-180	
45	*Stephania tetrandra* (Menispermaceae)	BGC-823-180	34 and 35
		HCT116-260	
		Hep G2-210	
		A549-160	
46	*Piper arborescens* (Piperaceae)	KB-140	36 and 37
		A549-180	
		P388-180	
		HT29-260	
47	*Plumbago zeylanica* L. (Plumbaginaceae)	MG63-160	38 and 39
		MCF7-230	
48	*Nigella sativa* (Iridaceae)	PC3-300	40
49		LL/2-260	
50		HeLa-280	
51	*Cyrtanthus contractus* (Amaryllidaceae)	HeLa-200	41
		MCF7-290	
		A431-260	
52	*Capsicum annuum* (Solanaceae)	HCT LoVo-250	42
		MCF7-200, MDA-MB231-240	
		LNCaP-180	
		HL-60-255	
		PANC1-200	
53	*Broussonetia papyrifera* (L.) (Moraceae)	BEL-7402-185	27
		Hela-150	
54	*Narcissus jonquilla* (Amaryllidaceae)	PC3-290	43
		LoVo-300	
		A549-350	
		MCF-7-380	
55	*Hymenocallis littoralis* (Amaryllidaceae)	PANC1-250	
56		MV4-11-110	44
57		U87-160	
58		MCF7-145	
59		OVCAR3-135	
60		Hep G2-230	
61		PANC1-280	
62		U87-260	

Berberine (1) and its seven synthetic isomers having different substituents, such as [propyl benzene (1), 1-chloro-4-ethylbenzene (2), butane-1,1-diyldibenzene (3), 4,4-diphenylbutyl (4), phenyl-pentyl (5), phenyl-hexyl (6), 5,5-diphenyl-amyl (7) and 6,6-diphenyl-hexyl (8)], were screened for prostate cancer, lung cancer, liver cancer, and chondrosarcoma and these were found to work in a variety of ways to prevent cancer. It inhibited cyclin D1 and E1 in lung cancer and CDK4 expression and modulating cyclin D1 in colorectal cancer and hepatoma cancer. Berberine upregulated the level of p53 and p21 in chondrosarcoma by regulating the PI3K/Akt and p38 signaling pathways.^45^

Evodiamine (9) and its six derivatives with varied substituents [4-chlorobenzoyl (10), 12-chloroevodiamine (11), 3-fluoroevodiamine (12), 10-hydroxyevodiamine (13), 3-fluoro-10 hydroxyevodiamine (14) and 3-amino-10-hydroxyevodiamine (15)] were screened for the treatment of colon cancer, osteosarcoma, pancreatic carcinoma, prostate cancer, leukemia and breast cancer. By inhibiting the caspase inhibitor evodiamine inhibits cervical cancer. Caspase inhibition causes alteration in Bax and Bcl-2 balance, which decreases apoptosis.^46^ It suppresses the liver cancer by inducing apoptosis and inhibiting the PI3K/Akt pathway.^47^

Matrine (16) and its five derivatives with varying substituents, such as [11-(3-methylbenzyl)dodecahydro-1*H*,5*H*,10*H*-dipyrido[2,1-*f*:3′,2′,1′-*ij*][1,6]naphthyridin-10-one (17), 4-methyl-10-oxotetradecahydro-1*H*,5*H*-dipyrido[2,1-*f*.3′,2′-*ij*][1,6]naphthyridin-4-ium iodide (18), (11(*Z*)-11-(6-bromonaphthalen-2-yl)(hydroxy)methylene)dodecahydro-1*H*,5*H*,10*H*-dipyrido[2,1-*f*:3′,2′,1′-*ij*][1,6]naphthyridin-10-one (19), 11-(hydroxy(naphthalen-2-yl)methyl)dodecahydro-1*H*,5*H*,10*H*-dipyrido[2,1-*f*:3′,2′,1′-*ij*][1,6]naphthyridin-10-one (20) and 11-(hydroxy(6-methoxynaph-thalen-2-yl)methyl)dodecahydro-1*H*,5*H*,10*H*-dipyrido[2,1-*f*:3′,2′,1′-*ij*][1,6]naphthyridin-10-one (21)], showed anticancer potential against lung cancer, breast cancer, liver cancer, prostate cancer, leukemia and sarcoma. It causes caspase-mediated cell death in lung cancer by impeding the G_1_/G_0_ phase of the cell cycle.^48^ Matrine showed anticancer activity against pancreatic cancer by inducing ROS generation, and induced death.^49^ Crinine-type (22) alkaloids and their fourteen derivatives [haemanthamine (23), haemanthidine (24), vitattine (25), hydroxyvitattine (26), crinamine (27) 11-*O*-acetylcrinamine (28) 11-*O*-methylcrinamine (29), 6-hydroxycrinamine (30), hamayne (31), 3-*O*-acetylhamayne (32), 8-*O*-demethylmaritidine (33), papyramine (34), dihydrocrinamine (35) and dihydrohamayne (36)] are also promising therapeutic candidates for the treatment of apoptosis-resistant tumors, particularly glioblastoma. The crinine-type alkaloid inhibits glioblastoma cell proliferation *via* cytostatic effects resulting from the rigidification of the actin cytoskeleton. Bulbispermine (37) showed anticancer activity against glioblastoma and leukemia by inhibiting apoptosis resistance.^25^ Distichamine (38) in leukemia alter the cell cycle and induce death by activating the caspase 3 and 7.^50^ lycorine (39) and amarbellisine (40) reduce Mcl-1 at the translational level, which causes cell death in leukemia cells. Lycorine promotes the intrinsic apoptotic cascade in bladder cancer by decreasing the PI3K–Akt pathway and boosting the expression of the PTEN protein, which acts as a negative regulator of p-Akt.^51^ ungeremine (41) showed cytotoxic effects against leukemia by inhibiting cell proliferation through caspase activation, matrix metalloproteinases (MMP) modification, and also increasing ROS production.^52^ Piperine (42) inhibits cell proliferation by activation of apoptotic signalling pathways, modulation of ER stress and induction of detoxification of enzymes.^53^ Sanguinarine (43) and its one derivative dihydrosanguinarine (44) showed their anticancer potential by suppressing the abnormally active signal transduction pathways, cell apoptosis, and cancer cell proliferation.^54^ Tetrandrine (45) showed anti-cancer properties against lung, colon, bladder prostate, and many more, as shown in Table 1. Tetrandrine's anticancer properties may be linked to autophagy, cell cycle arrest, alleviate metastasis and suppression of tumor cell proliferation.^55^ Piplartine (46) caused G2/M cell cycle arrest, followed by mitochondrial-dependent apoptosis, as shown by chromatin condensation and inter-nucleosomal DNA breakage.^56^ Plumbagin (47) showed its anticancer potential through the NF-k, STAT3, and Akt regulatory signaling pathways. It was also a potent ROS inducer, a suppressor of cellular glutathione, and a novel proteasome inhibitor generating DNA double-strand breaks *via* oxidative DNA base damage.^57^

Thymoquinone (48) and its two derivatives [thymoquinone (49) and dithymoquinone (50)] have anti-cancer properties through a variety of mechanisms, including selective antioxidant activity, DNA structural interference, effects on carcinogenic signaling molecules/pathways, and immunomodulation.^58^ Narciprimine's (51) effects on DNA topoisomerase have also been studied. The findings demonstrated that narciprimine was dose-dependently efficacious in DNA topoisomerase processes. The potential of this alkaloid to interfere with topoisomerase was somewhat associated with anticancer activity measured in HeLa, MCF-7, and A341 cells.^41^ Multiple mechanisms were involved in capsaicin's (52) anticancer activity, including increased intracellular calcium, inhibition of p53, STAT3 and nuclear factor B.^59^ Norchelerythrine (53) works as an anticancer agent by various methods, including apoptosis, inhibiting aromatase, disrupting tubulin aggregation, inhibiting topoisomerase, and inhibiting ER.^60^ In the prostate and breast cancer cells, narciclasine (54) causes inactivation of mitochondrial membrane potential, cytochrome release and caspase activations.^27^ Pancratistatin (55) and its seven synthetic analogues with varied substituents [JCTH-1 (56), JCTH-2 (57), JCTH-3 (58), JCTH-4 (59), SVTH-5 (60) SVTH-6 (61) and SVTH-7 (62)] inhibit tumor xenograft growth by disrupting mitochondrial activity and by activating the intrinsic apoptotic pathway. SVTH-7 inhibits mitochondrial complex II and III, reducing pro-apoptotic effects on cancer cells and on mitochondria.^44^

### 3.1 *In vivo* anti-cancer studies of alkaloids

In the drug development process, preclinical data give complete information, including preliminary efficacy, toxicity, pharmacokinetics, and safety of potential lead compound. This information can be used to determine whether or not a compound should be pursued further for clinical trials. In this context, various *in vivo* studies reporting the anti-cancer evaluation of alkaloids have also been summarised as follows:

Berberine anticancer activity against colorectal cancer was tested in a xenograft model of BALB/c nude mice. Mice were injected with KM12C cell sublines, shCtrl, and shRXR. After the tumor had grown, the infected mice were given berberine (10 mg kg^−1^). Berberine reduced the length of the tumor which could be due to the induction of nuclear-catenin degradation, significantly reducing endogenous c-Cbl, Ki67, Cdc2, c-Myc, and CIP1. Berberine also shows its activity by inhibiting the β-catenin signaling pathway.^61^ A xenograft model was used to test berberine's anticancer activity against endometrial cancer. Mice were injected with HEC-1-A. When the tumor had grown, mice were divided into three groups. Groups were given either 0.5% MC (vehicle control) or berberine (50 mg kg^−1^, p.o.qd or 100 mg kg^−1^, p.o.qd), orally. Berberine treatment significantly reduced the invasion of HEC-1-A cells at 50 mg kg^−1^ and 100 mg kg^−1^,^62^ and in lung cancer, it showed its potential at 200 mg kg^−1^ and 25 mg kg^−1^ in nude mice.^63^ The anticancer activity of matrine against lung cancer was tested in a xenograft model of BALB/c nude mice by inserting the LA795 cell. The infected mice were given matrine (80 mg kg^−1^) and a vehicle. It reduced the length of the tumor by regulating transmembrane protein 16A.^64^ Matrine anticancer activity in breast cancer was investigated by inserting the C57BL cell subcutaneously. Then 50 mg per kg matrine was injected once a day at an early stage of cancer. Mice were forfeited after 21 days. The tumor was collected and evaluated. The results indicated that matrine reduced breast cancer angiogenesis by inhibiting the Wnt/β-catenin signaling pathway.^65^ Piperine anticancer activity against breast cancer was investigated in BALB/c mice. Then, 2 × 105 EEMT6/P cells were injected subcutaneously. Following this, 25 mg per kg matrine was injected once a day at an early stage of cancer. Mice were forfeited after 14 days. The tumor was collected and evaluated.^66^ Lycorine anticancer activity against prostate cancer was tested in a xenograft model of BALB/c nude mice by inserting the RM-1 cells. After the tumor had grown to about 20 mm^3^ in diameter, the infected mice were given lycorine (10 mg kg^−1^) and a vehicle. It shows its anticancer potential by inhibiting the p65 and IKK-β phosphorylation, downregulating the Ki-67 expression and increasing caspase 3 in tumor tissue.^67^ Lycorine anticancer activity against liver cancer was tested in a xenograft model of Kunming mice. Then, 5 × 106 of H22 cells were injected into the axillary region of the right fore limb. The infected mice were given lycorine (10 mg kg^−1^, 20 mg kg^−1^ and 40 mg kg^−1^) and a vehicle. Lycorine reduced the length of the tumor in a dose-dependent manner.^68^ Evodiamine was tested for anticancer activity against tongue cancer in a xenograft model of BALB/c nude male mice. For 35 days, infected mice were given evodiamine (10 mg kg^−1^) intraperitoneally. It reduced the tumor length by regulating the NF-B pathway.^69^ Evodiamine was tested for anticancer activity against lung cancer in a xenograft model of BALB/c nude female mice. For 22 days, the infected mice were given evodiamine (20 mg kg^−1^) *via* gavage. Evodiamine reduced the tumor length by increasing CD8 + T cells and decreasing the MUC1-C/PD-L1 axis.^70^ The anticancer activity of evodiamine against lymphoma was tested in a KM male mouse xenograft model. For 21 days, infected mice were given evodiamine (20 mg kg^−1^) *via* gavage three times a day. Evodiamine shortened the tumor length by downregulating Ki-67 expression.^71^ Evodiamine was tested for anticancer activity against colorectal carcinoma in a xenograft model of BALB/c nude female mice. For 22 days, infected mice were given evodiamine (10 mg kg^−1^, i.p.). Evodiamine shortened the tumor's length by suppressing hypoxia-inducible factor 1-α-mediated angiogenesis.^72^ The anticancer activity of evodiamine against lung cancer was tested in a xenograft model SCID nude mice. For 14 days, the infected mice were given evodiamine (20 mg kg^−1^) *via* gavage. Evodiamine showed its potential by inhibiting heat shock protein.^73^

**Fig. 5:**
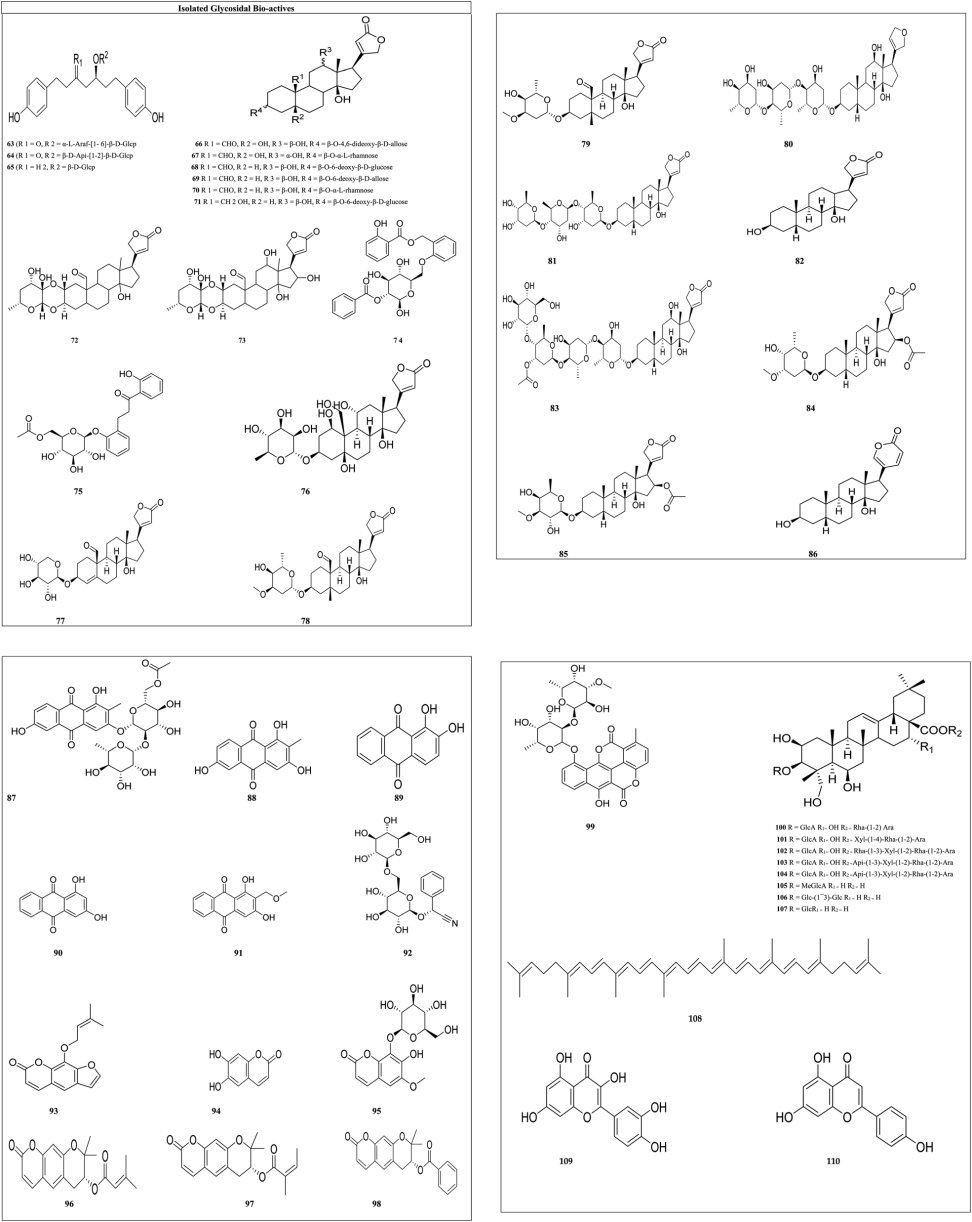
Structures of isolated glycosides.

Glycosides: the secondary metabolites, which produce at least one sugar fraction as well as one non-sugar fraction on hydrolysis, are termed glycosides. These include bufalin, antiaroside, papyriferoside, calotropin, ouabain, hyrcanoside, and many more glycosides. The antiproliferative activity of cardiac glycosides has attracted a lot of attention, because the sugar fraction increased solubility and their stereochemistry affected the binding affinity of the receptor protein.^74^ Various isolated glycosides IC_50_ values with their cytotoxity on different cell lines are reported in Table 2, and the structures of the isolated glycosides are shown in Fig. 5.

**Table 2:** Various isolated glycosides, their biological sources and reported IC_50_ values against different types of cancer cell lines

Isolated compound	Glycoside (biological source)	Cancer cell line along with IC_50_ (nM) value	References
63	*Betula papyrifera* (Betulaceae)	A-549-50	75
64		DLD-1-90	
65		WS1-60	
66	*Antiaris toxicaria* (Moraceae)	KB-150	76
67		1A9-190	
68		CAKI-1-130	
69		S-KMEL-2-200	
70		KB-250	
71		S-KMEL-2-320	
72	*Asclepias subulata* (Apocynaceae)	A549-180	77
		LS 180, 147	
73		PC-3-90	
74	*Salix acmophylla* (Salicaceae)	MCF7-184	78
75		NCI-H460-210	
76	*Strophanthus gratus* (Apocynaceae)	A549-12.66	79
		HCT116-10.44	
		PANC1-42.36	
		Hela-22.6	
77	*Coronilla varia* (Fabaceae)	HCT116-144	80
78		MCF-7-165	
79		U-2 OS-44	
80	*Digitalis purpurea* (Plantaginaceae)	Hela-25.44	74
81	*Digitalis purpurea* (Plantaginaceae)	GSC-22	
82	*Digitalis purpurea* (Plantaginaceae)	U2OS-18	
		SaOS2-15	
83	*Digitalis purpurea* (Plantaginaceae)	Huh7-22	
		Mahlavu-19	
84	*Digitalis purpurea* (Plantaginaceae)	DAOY-50	
85	*Digitalis purpurea* (Plantaginaceae)	U2OS-95	
		SaOS2-90	
86	*Bufo melanostictus* Schneider (Bufonidae)	MDA-MB231-20	81
		Hela-16.6	
		SW620 15.6	
		A549-15.57	
87	*Rubia philippinensis* (Rubiaceae)	MCF7-240	82
88		SK-MEL5-175	
89		SK-MEL5-235	
90	*Rubia philippinensis* (Rubiaceae)	B16 F10-80	
91	*Rubia philippinensis* (Rubiaceae)	MCF-7-178	
92	Amygdalin, *Amygdalus communis* (Rosaceae)	TCCSUP-22.8	83
		HeLa-16.8	
		SNU-C4-34.8	
93	*Angelica archangelica* (Apiaceae)	HepG2-39.34	84
		SPC-A1-80	
		SGC-7901-160	
		HeLa-52.86	
		K562-183	
94	*Artemisia capillaris* (Asteraceae)	HN22-50.34	85
		HSC4-20.24	
95	*Fraxinus rhynchophylla* (Oleaceae)	Hep3B-19.34	86
96	*Ferulago campestris* (Apiaceae)	A549-29.34	87
97	*Ferulago campestris* (Apiaceae)	A549-180.4	
98		A549-205.4	
99	*Streptomyces chartreusis* (Streptomycetaceae)	L1210-20	88
		P388-70	
		B16-90.34	
100	*Vitellaria paradoxa* (Sapotaceae)	HL60-30	89
101		A549-170	
103		AZ521-78	
104		SKBR-3120	
105		AZ521-108	
106		HL60-90	
107		A549-270	
108	*Solanum lycopersicum* (Solanaceae)	HT-29-70.89	90
109	*Malus pumila* (Rosaceae)	HeLa-70.12	91 and 92
		AGS-40	
		A549-50	
		HepG2-13.16	
110	*Brassica napus* (Brassicaceae)	PC3-100.9	93
		HCT116-360	
		NCIH929-100.73	

Papyriferoside (63) and its two derivatives with different substitutions [(R_1_ = O, R^2^ = α-l-araf-[1-6]-β-d-Glcp) (63) (R_1_ = O, R^2^ = β-d-Api-[1-2]-β-d-Glcp) (64) (R_1_ = H_2_, R^2^ = β-d-Glcp) (65)] show cytotoxic effects against lung cancer, colorectal cancer, and normal skin cancer by inducing apoptosis, resulting in cell cycle arrest, downregulation of IB phosphorylation and BCL-2, and over expression of cleaved caspase and BAX proteins.^94^ Antiaroside (66) and its five derivatives with varied substituents [(R^1^ = CHO, R^2^ = OH, R^3^ = α-OH, R^4^ = β-*O*-α-l-rhamnose) (67), (R^1^ = CHO, R^2^ = H, R^3^ = β-OH, R^4^ = β-*O*-6-deoxy-β-d-glucose) (68), (R^1^ = CHO, R^2^ = H, R^3^ = β-OH, R^4^ = β-*O*-6-deoxy-β-d-allose) (69), (R^1^ = CHO, R^2^ = H, R^3^ = β-OH, R^4^ = β-*O*-α-l-rhamnose) (70) and (R^1^ = CH_2_OH, R^2^ = H, R^3^ = β-OH, R^4^ = β-*O*-6-deoxy-β-d-glucose) (71)] suppress lung cancer cell proliferation by inhibiting the cell migration and the epithelial–mesenchymal transition (EMT) processes.^95^ Calotropin (72) and its derivative 12,16-dihydroxycalotropin (73) induce cell death through an apoptotic process that is caspase-dependent and ideally driven by an extrinsic pathway. These *A. subulata* cardenolide glycosides could be used as anticancer drugs. Acmophyllin A (74) and Acmophyllin B (75) both promote apoptosis, damage DNA, and/or denature proteins, which trap free radicals and protect cellular macromolecules from oxidative mutilation.^96^ Ouabain's (76) administration causes an increase in programmed cell death, intracellular ROS production, and breakage of DNA strands. Ouabain also inhibited STAT3-mediated transcription and downstream target proteins, as well as suppressing STAT3 levels and phosphorylation.^79^ Hyrcanoside (77) and its two derivatives [deglucohyrcanoside (78) and cymarin (79)] showed anticancer potential against leukemia, lung adenocarcinoma, colorectal carcinoma, adenocarcinoma, breast carcinoma, and osteosarcoma by inducing cell cycle arrest in the G_2_/M phase.^80^ Phyto-compounds digoxin (80), digitoxin (81), digitoxigenin (82), lanatoside (83), oleandrin (84) and neritaloside (85), reported in Table 2 are cardiac glycosides. The cardiac glycoside binding site has been investigated, in what manner the multifunctional groups of sodium pump is blocked. The first extracellular subunit channel is the most critical component of the binding site. The −1 subunit is overexpressed in several cancers including lung cancer, renal carcinoma, glioma, and melanoma.^97^ Anti-proliferation, Na^+^/K^+^-ATPase activity targeting, and steroid receptor coactivator inhibitions were the key anti-cancer molecular mechanisms of bufalin (86).^81^ 2-Methyl-1,3,6-trihydroxy-9,10-anthraquinone 3-*O*-(6′-*O*-acetyl)-α-rhamnosyl(1 → 2)-β-glucoside (87), 2-methyl-1,3,6-trihydroxy-9,10-anthraquinone (88), alizarin (89), purpurin (90) and lucidin-ω-methyl ether (91) can cause cell death in CNE cells by arresting CNE cells at the G1 stage.^98^ Amygdalin (92) has also been demonstrated to prevent various cancer cells by reducing integrin expression and catenin levels, and inhibiting the Akt-mTOR pathway, which may contribute to cancer cell metastasis suppression.^99^ Imperatorin (93) decreases the viability of HeLa cells and laryngeal carcinoma (Hep-2) cells by inducing apoptosis and elevating the activity of apoptosis mediator's caspase-3 and caspase-8 in both cell lines.^84^ Esculetin (94) treats HN22 and HSC4 cells resulted in a substantial reduction of cancer cells, as well as the regulation of Sp1 regulatory protein.^85^ Fraxini's (95) anti-proliferative effect in Hep3 cells was related to apoptosis and alterations in the mitochondrial structure.^86^ Coumarin glycosides grandivittin (96), agasyllin (97) and aegelinol benzoate (98) have anticancer properties and showed their anticancer potential by lowering the mitochondrial depolarization potential, modulating the mitochondrial protein pathway, enhancing Bid, Bad, and Box protein expression, and lowering Bcl-xl and Mcl-1 expression.^100^ Chartreusin's (99) anticancer actions are due to DNA binding and inhibition of topoisomerase II.^101^ Paradoxoside (100) and its seven derivatives with disparate substituents [(R = GlcA R_1_ = OH R_2_ = Xyl-(1-4)-Rha-(1-2)-Ara) (101), (R = GlcA R_1_ = OH R_2_ = Rha-(1-3)-Xyl-(1-2)-Rha-(1-2)-Ara) (102), (R = GlcA R_1_ = OH R_2_ = Api-(1-3)-Xyl-(1-2)-Rha-(1-2)-Ara) (103), (R = GlcA R_1_ = OH R_2_ = Api-(1-3)-Xyl-(1-2)-Rha-(1-2)-Ara) (104), (R = MeGlcA R_1_ = H R_2_ = H) (105), (R = Glc-(1-3)-Glc R_1_ = H R_2_ = H) (106) and (R = GlcR_1_ = H R_2_ = H) (107)] showed anticancer activity on human leukemia, lung cancer, stomach cancer and breast cancer by regulating the microphthalmia-associated transcription factor (MITF), TRP-1 and TRP-2 expression.^89^ Lycopene^90^ (108) showed its potential against colon cancer by significantly elevated cleaved caspase 3, BAX, cleaved PARP, and 8-oxo-dG levels in cancer cells. Quercetin^91,92^ (109) diminished the viability of cervical cancer cells through the induction of G_2_/M phase cell cycle arrest and apoptosis, alongside the suppression of cell migration and invasion. In the context of gastric cancer, quercetin was observed to inhibit miR-143, while in HepG2 cells, p53 and miR-34a were found to be inhibited. Apigenin [110] showed its anticancer potential by inhibiting the STAT1/COX-2/iNOS signaling pathway.^93^

### 3.2 *In vivo* anti-cancer studies of glycosides

Recent pre-clinical studies of various glycosides from the natural sources with anti-cancer potential are summarised below:

Lycopene anticancer activity against ovarian cancer was evaluated in egg-laying hens. First, 200 mg kg^−1^ and 400 mg kg^−1^ of lycopene were given to the hens daily for 12 months. At the end of 12 months, hens were sacrificed and ovarian tissues and blood were collected and evaluated. By reducing the expression of NF-κB and STAT3 and increasing the expression of heme oxygenase 1, lycopene shows its anticancer potential.^102^ Lycopene consumption significantly reduced the metastatic load in an ovarian carcinoma-bearing rat model. Its consumption reduces the expression of CA125. The anti-proliferative and anti-metastatic effects were augmented by the down regulation of ITGB1, MMP9, ITGA5, FAK, ILK, and EMT markers, which reduced the MAPK activity and inhibited integrin 5 protein expression. Lycopene activity against tobacco-induced carcinogens was evaluated in male ferrets. For one month, six groups of ferrets were given 50 mg kg^−1^ of NNK to induce lung and liver lesions. Following the induction of the lesions, each group was given dietary lycopene for 26 weeks at doses of 2.2 and 6.6 mg kg^−1^ BW per day, respectively. Lycopene supplementation inhibited NNK-induced pulmonary α7 nAChR and hepatic CYP2E1, which were linked to lower mortality and occurrences of both pulmonary and hepatic lesions.^103^ The anticancer potential of quercetin against colon cancer was tested in 4 week-old Balb/C mice. The control group received no treatment, whereas the treatment group received 10 mg kg^−1^ of quercetin per day. The tumor volume was significantly reduced in the treatment group. According to the findings, quercetin has anticancer properties by inhibiting the expression of Notch-1, Jagged 1, Hes-1, and Presenilin-1.^104^ Then 2 × 105 MCF-7 cells were inserted into mice. Two groups were divided simultaneously; one group was the untreated group which receive only vehicle while the second group received quercetin (50 mg kg^−1^ i.p.) twice a day for a month. Quercetin inhibits tumor by downregulation of VEGF, PKM2, beclin-1, and p-Akt/Akt.^105^ Apigenin's anticancer activity against chondrosarcoma was investigated in athymic nude mouse xenografts. Then, 2 × 105 Sw1353 cells were inserted into mouse. The untreated group received no treatment, while the treatment group received 5 mg kg^−1^ apigenin daily. In the treatment group, the tumor volume was significantly reduced. Apigenin has anti-cancer properties because it inhibits Ki67 expression. Apigenin-induced cell cycle arrest and apoptosis by regulating the expression of Bcl-2.^106^ Additionally, apigenin (3 mg kg^−1^) inhibited NSCLC xenograft growth and metastasis by targeting the dipeptidyl peptidase IV (DPPIV) enzyme.^107^ Digoxin anticancer activity against human lung cancer was investigated in BALB/c nude mouse xenograft model. Following this, 1 × 107 A549 cells were implanted in mouse. After the tumor had grown to about 100 mm^3^ in diameter, the infected mice were daily given digoxin (1.0 mg kg^−1^). After 14 days, mice were forfeited, and the tumor volume was collected and evaluated. The results indicated that digoxin inhibits lung cancer by inhibiting both DNA DSB and SSB repairs.^108^ Digitoxin anticancer activity against cervical cancer was investigated in a BALB/c nude mouse xenograft model. Then, 5 × 106 HeLa cells were implanted into mouse. After the tumor had grown to about 300 mm^3^ in diameter, the infected mice were given digitoxin (1.0 to 2.0 mg kg^−1^) daily. After 19 days, mice were forfeited, and the tumor volume was collected and evaluated. Digitoxin shows its potential by arresting the cell.^109^ Bufalin anticancer activity against human lung cancer was investigated in a BALB/c nude mouse xenograft model. Then, 8 × 106 A549 cells were implanted into mouse. After the tumor had grown to about 300 mm^3^ in diameter, the infected mice were given bufalin (1 mg to 6 mg kg^−1^) daily. After 19 days, mice were forfeited, and the tumor volume was collected and evaluated. Bufalin shows its potential by activation of caspase-3 and the cleavage of PARP in A549 cells.^110^ Bufalin anticancer activity against breast cancer was investigated in athymic nude mice. Then, 5 × 106 MB-231 cells were injected subcutaneously into both dorsal regions of mice and 10 μl of bufalin was injected once a day at an early stage of cancer. Mice were forfeited after 21 days. The tumor was collected and evaluated. The results indicated that bufalin reduced breast cancer angiogenesis by inhibiting the MAPK and NF-kB pathways.^111^ Alizarin anticancer activity against pancreatic cancer was investigated in the mouse xenograft model. Then 5 × 106 MIA PaCa-2-luc cells were implanted into the mouse. After the tumor had grown to about 300 mm^3^ in diameter, the infected mice were given alizarin (10 to 30 mg kg^−1^) daily. After 19 days, mice were sacrificed, and the tumor volume was collected and evaluated. Digitoxin showed its potential by abrogating NF-κB activation.^112^

#### Miscellaneous bio-actives

**Fig. 6:**
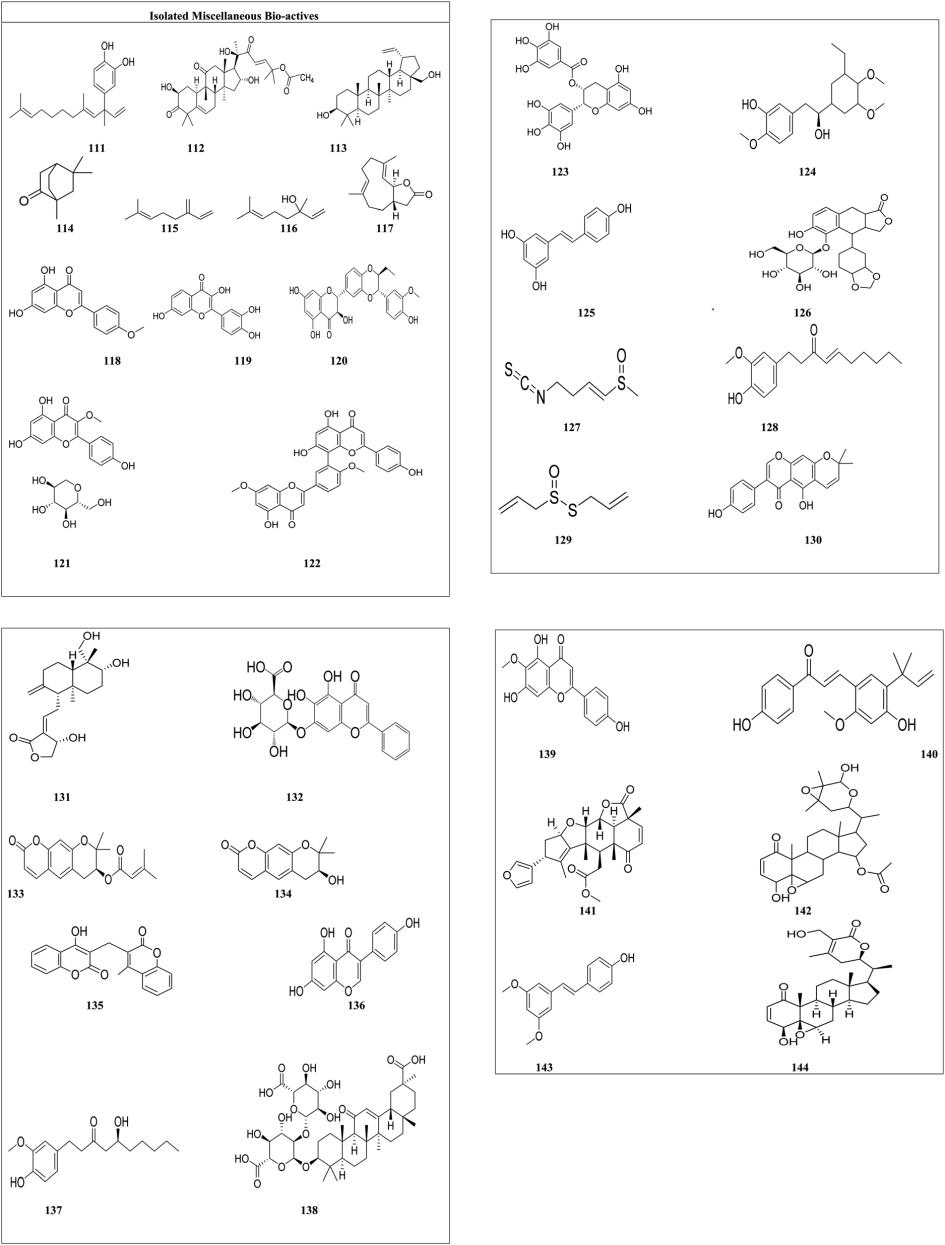
Structures of isolated miscellaneous drugs.

Various plant bio-actives other than glycosides and alkaloids also possess the anticancer potential and showed significant activity against various types of cancers. These secondary compounds may contain terpenoids, terpenes, flavonoids, and lignans. Table 3 presents the various secondary metabolites exerting cytotoxicity on different cell lines, and the structures of miscellaneous bioactives are shown in Fig. 6.

**Table 3:** Different bio-actives, their biological sources and IC_50_ values in different cancer cell lines

Isolated compounds	Biological source	Cancer cell line along with IC_50_ (nM) value	References
111	*Pothomorphe umbellata* (Piperaceae)	SK-MEL2-95	113
		SK-MEL103-100	
		SK-MEL147-90	
112	*Cucumis sativus* (Cucurbitaceae)	SK MEL28-45	114
		A-375-30	
113	*Betula pendula* (Betulaceae)	SK-MEL 28-200	115
		MSK-MEL2-198.4	
		G361-190	
114	*Cinnamomum camphora* (Lauraceae)	A549-18.7	116
115	*Cannabis sativa* (Cannabaceae)	A549-120	117
116	*Cannabis sativa* (Cannabaceae)	C6-22	
117	*Colchicum autumnale* (Colchicaceae)	HA22T/VGH-90	118
		OVCAR3-129	
		T24-180	
		MDA-MB-231-30	
118	*Robinia pseudoacacia* (Fabaceae)	LOVO-100	119
119	*Malus domestica* (Rosaceae)	Mel 928-25	120
120	*Silybum marianum* (Asteraceae)	DU145-24	121 and 122
		MDAMB-468-46	
		MMP2-63.5	
		CD34-56.4	
121	*Anaphalis neelgerriana* (Asteraceae)	HaCaT-28	123
		HL-60-40	
		A549-68	
		H1299-64	
122	*Ginkgo biloba* (Ginkgoaceae)	MCF7-48, T47D-52	124

**Polyphenols**			
123	*Camellia sinensis* (Theaceae)	A375 12.8	125
		Hs294T-8.78	
124	*Combretum caffrum* (Combretaceae)	P388-28	36
125	*Cajanus cajan* (Fabaceae)	HepG2-50.99	126
		MCF-7-20.56	
		A549-60.18	

**Lignan**			
126	*Justicia hyssopifolia* L. (Acanthaceae)	MALME-3M-16	127
		SK-MEL-5.32	
		UACC257-48	

**Isothiocyanate**			
127	Sulforaphene	MCF-7-41.1	128
		HepG2-40.0	
		HT-29-42	
128	*Zingiber officinale* (Roscoe)	HeLa-250.68	129
		SiHa-370.52	
129	*Allium sativum* (Amaryllidaceae)	HepG2-19.26	130
		MCF7-28.51	
		A549-36	
		PC3-77.92	
130	*Derris eriocarpa* (Leguminosae)	KB-40.13	131
		P-388-34.31	
		H2108-56.5	
131	*Andrographis paniculata* (Acanthaceae)	HL 60-20.4	132
		HepG2-40.2	
		Lovo-8.6	
132	*Scutellaria baicalensis* (Lamiaceae)	MDA-MB-231-34.77	133
		MCF7-41.78	
133	*Angelica gigas* (Apiaceae)	SNU-216-50	134
		HT29-293.064	
		A549-200	
		B16F10-80	
134	*Angelica gigas* (Apiaceae)	PC3-36.3	135
135	*Melilotus officinalis* (Fabaceae)	MCF7-40	136
136	*Glycine max* (legumes)	MCF7-15	137
		HepG2-25	
		NCI-H1299-55	
137	*Zingiber officinale* (Roscoe)	S-180-19.18	138
		HL-60-111.4	
138	*Glycyrrhiza glabra* (Fabaceae)	MDA-MB-231-84.22	139
139	*Salvia involucrata* (Lamiaceae)	MCF-7-25.44	140
		HCC38-65.42	
140	*Glycyrrhiza glabra* (Fabaceae)	MCF-7-17.63	141
		A549-11.55	
		DU-145-9.45	
141	*Azadirachta indica* (Meliaceae)	EJ-30	142
		MDA-MB-231-10.97	
		HT29-40	
		HCT116-75	
142	*Physalis pubescens* L. (Solanaceae)	SKOV3-60.63	143
143	*Polygonum cuspidatum* (Polygonaceae)	HeLa-30	144
144	Withaferin A	Panc1-10.24	145
		BxPc-320.78	
	*Withania somnifera* (Solanaceae)	U87MG-10.4	
		GBM2-19	

In melanoma cell lines, 4-nerolidylcatechol (111) is reported as an inhibitor of cell invasiveness, owing to the G1 cell cycle arrest and inhibition of MMP-2 activity.^113^ Melanoma has a high prevalence of B-RAF mutations. Cucurbitacin B (112) could be a possibility for inhibiting the signaling kinase pathway. Cucurbitacin B is a kinase inhibitor for B-RAF and MEK1.^114^ Betulin's (113) anticancer action is based on the stimulation of apoptotic cell death. Betulin treatment caused cytomorphological changes that are typical of apoptotic cells, including cell rounding and the production of apoptotic bodies.^115^ Camphor (114) white oil caused transcriptional alterations in immune-related genes identified by RNA-sequencing *in vivo*, leading to tumor regression mediated by cytotoxic T cells.^116^ The cytotoxicity of myrcene (115) against leukemia cells was shown to be substantial. At 0.01 g ml^−1^, myrcene decreased *t*-butyl hydroperoxide-induced DNA damage in human B lymphoid NC–NC cells by 50%.^146^ Linalool (116) inhibited mitochondrial complexes I and II, increased reactive oxygen species, and lowered ATP and GSH levels in HepG2 cells. Linalool also upregulated p53 and cyclin-dependent kinase inhibitors, which induced strong apoptosis in a variety of leukemia cells.^147^ By decreasing the mRNA and protein expression of human telomerase reverse transcriptase, costunolide (117) inhibited proliferation in human B cell leukemia cells^148^ acacetin (118) inhibited epidermal growth factor (EGF)-induced cell transformation and phosphorylation of p70S6K. Acacetin binds to the p110 subunit of PI3-K, interacting with Val828, Glu826 and Tyr813 residues.^119^ Mitf, a transcription factor related to microphthalmia and found downstream of the Wnt/-catenin pathway, has emerged as a key melanoma prognostic factor. Fisetin (119) (3,7,3′,4′-tetrahydroxyflavone) treatment of melanoma cells resulted in decreased cell survival, G_1_-phase arrest, and inhibition of Wnt/-catenin signaling. Fisetin-treated cells have higher intracellular levels of Axin and TrCP, as well as reduced glycogen synthase kinase 3 phosphorylation and catenin stabilization.^120^ Silymarin (120) blocks cyclin-dependent kinase (CDK) activity and increases the levels of the CDK inhibitors p21CIP1 and p27KIP1 such that they are more tightly bound to CDKs, which suppresses EGFR signaling. Silymarin inhibits development at the G1 and G2 checkpoints.^121^ The bioactive flavonoid, astragalin (121) heptaacetate (AHA) promotes apoptosis in HL-60 cells by releasing cytochrome c into the cytoplasm. Activation of Bax, caspase-3/-7, and p38MAPK, as well as intracellular ROS production and suppression of cell signaling pathways JNK/SAPK and ERK 1/2 also promote apoptosis in HL-60. TNF-induced NF-B activation is significantly inhibited by astragalin in A549 and H1299 cells. Furthermore, astragalin-induced cell death is associated with a time- and dose-dependent increase in the Bax/Bcl-2 ratio, as well as increased cleavage of caspase-3/-9 and PARP^123^ Ginkgetin (122) decreased cell viability in breast cancer and blocked estrogen receptor (ER) expression at mRNA and protein levels. Ginkgetin therapy also reduced the expression of survivin, and cyclin D1, which are also ER targets.^124^ Epigallocatechin gallate (123) inhibited cell proliferation by reducing the PCNA protein level and promoted apoptosis in melanoma by assessed cleavage of PARP, TUNEL assay. Treatment of melanoma cells with epigallocatechin gallate leads to a reduction in cyclin D1 and cdk2 protein levels, as well as stimulation of the cyclin kinase inhibitors (ckis) and p27KIP1.^125^ Combretastatin (124) is the new molecule of vascular disrupting medicines that target tumor blood channels and prevent angiogenesis. Combretastatin affects DNA structure and function by interfering with nucleic acid production and transcription and inhibiting cell proliferation.^149^ Resveratrol (stilbenoid) [125] was observed to halt the cell cycle at the G_2_/M phase, also elevating intracellular reactive oxygen species (ROS) and caspase 3 activity, and increasing the Bax/Bcl-2 protein ratio, all of which are indicative of apoptosis in hepatic cancer.^126^ Elenoside (126) was screened for its anticancer potential on skin cancer cell lines but its mechanism of action is not known.^150^ Sulforaphane^128^ (127) induced mitochondrion-mediated apoptosis in cancer cells through the activation of caspase-9, followed by the cleavage and subsequent activation of caspase-3 and caspase-7. 6-Shogaol^129^ (128) has demonstrated the capability to inhibit the proliferation and migration of cervical cells through the suppression of the PI3K/Akt/mTOR signaling pathway. Allicin^130^ (129) has been shown to exert its cytotoxic effects by targeting cancer cells during the S and G_2_/M phases of the cell cycle. Alpinumisoflavone^131^ (130) modulates several signaling pathways, including PI3K/Akt, MAPK, and those regulating endoplasmic reticulum (ER) stress, ultimately leading to cell death and showcasing its therapeutic potential. Andrographolide^132^ (131) acts against leukemia by inducing cell cycle arrest in the G_0_/G_1_ phase, while also affecting the G_2_/M, G_1_, and S phases in hepatoma and colon cancer. The anti-tumor effects of baicalein^133^ (132) in breast cancer may be attributed to a novel mechanism involving tumor-associated macrophages. Decursin^134^ (133) reveals its potential by disrupting multiple signaling pathways; for instance, in gastric cancer, it alters the STAT3/c-My pathway and the MAPK/ERK1/2 pathways associated with colon and melanoma cancers. Additionally, decursin affects the PERK/ATF4 pathway, which plays a role in lung cancer. Decursinol^135^ (134) exerts its cytotoxic properties through the regulation of the G_0_/G_1_ phase in prostate cancer cells. The anticancer properties of dicumarol^136^ (135) have been linked to the inhibition of NQO1. Genistein^137^ (136) directly inhibits the PLK1 signaling pathway, demonstrating its anticancer efficacy. Gingerol^138^ (137) has the capacity to induce the generation of reactive oxygen species (ROS) in chronic (K562) and acute myeloid leukemia (U937) tumor cell lines, resulting in the disruption of the G2/M cell cycle, a reduction in cell cycle protein expression (including cyclin B1, Cdk1, Cdc25B, and Cdc25C), and alterations in cellular oxidant status that promote mitochondrial ROS production. Glycyrrhizin^139^ (138) offers protective and detoxifying effects by reducing the generation of reactive oxygen species, preserving glutathione (GSH), and differentially modulating apoptosis, as well as the Akt, ERK, and JNK pathways within the MAPK signaling cascade. Hispidulin^140^ (139) has been shown to inhibit TGF-β1-induced Smad2/3 signaling and cell migration across breast cancer. Licochalcone A^141^ (140) modulates the expression of various signaling pathways, including the EGFR/ERK, PI3K/Akt/mTOR, p38/JNK, MKK4/JNK, mitochondrial apoptosis pathway and the death receptor pathway. It inhibits the expression of proteins involved in the cell cycle and angiogenesis, and regulates both autophagy and apoptosis in cancer cells. Nimbolide^142^ (141) blocks the attainment of cancer hallmarks such as sustained proliferation, evasion of apoptosis, invasion, angiogenesis, metastasis, and inflammation by influencing kinase-driven oncogenic signaling pathways and shows its potential. Furthermore, physapubescin B^143^ (142) inhibited the transcriptional activity of STAT3, an oncogenic transcription factor implicated in numerous human malignancies, including ovarian cancer. Pterostilbene^144^ (143) was linked to the induction of apoptosis in tumor cells, as well as the downregulation of the oncogene E6 and the upregulation of activated caspase-3 levels. Withaferin A^145^ (144) was found to induce apoptosis and inhibit growth in pancreatic cancer cells through mitochondrial dysfunction and inactivation *via* the PI3K/Akt pathway.

### 3.3 *In vivo* anti-cancer studies of miscellaneous bio-actives

The pre-clinical data of various miscellaneous bio-actives including different terpenoids, flavones, polyphenols, *etc.*, are included as follows:

Epigallocatechin anticancer activity against lung cancer was evaluated in A/J female mice. Mice were injected with cisplatin for the induction of cancer. Epigallocatechin (1 mg ml^−1^, orally) was given to the mice. Male db/db mice were given tap water containing 40 ppm DEN for two weeks, followed by 34 weeks of drinking water containing 0.1% epigallocatechin gallate. The fortified drinking water containing epigallocatechin gallate significantly reduced the development of liver cell adenomas compared to the EGCG-untreated control group. In the livers of experimental mice, epigallocatechin gallate inhibited the phosphorylation of the ERK (extracellular signal-regulated kinase), Akt, Stat3, and JNK proteins. Chitosan-based nano-formulation of epigallocatechin gallate (10 mg ml^−1^) was also developed for the treatment of prostate cancer and the same was evaluated in a xenograft athymic nude mouse model. The formulation decreased the expression of Ki-67 and VEGF (markers of angiogenesis) in tissues of treated mice.^151^ Emodin's anticancer activity against human lung epithelial cancer was investigated in BALB/c nude mice with 50 mg per kg emodin daily, which inhibits cell growth (A549) by inducing ER-dependent apoptosis. In hepatocellular cancer, emodin shows its potential by inhibiting the p-JNK expression and increasing ERK and p38 phosphorylation. Emodin's anticancer activity against breast cancer was investigated in C57BL/6 and BALB/c mice. Then, 2 × 105 EO771 or 4T1 cells were inserted into mice and 40 mg per kg emodin was injected once a day at an early stage of cancer. Mice were sacrificed at different time intervals and the tumor was collected and evaluated. The results indicated that emodin reduced breast cancer angiogenesis by inhibiting M2 polarization and macrophage infiltration and increasing T-cell activation.^152^ Baicalein's anticancer activity against colon cancer was investigated in mouse xenograft. Then 50 mg kg^−1^ of baicalein was given to the infected (HCT116 cell) mouse. Baicalein shows its activity by downregulating the mitogen-activated protein kinase (MAPK) and p38 signaling pathways.^153^ A nude mouse model was used to test withaferin A's anticancer activity against colorectal cancer. CRC cells were inserted into the mouse. The mouse was given 5 mg per kg withaferin A orally after the onset of cancer. According to the findings, withaferin A has the potential to inhibit Akt overexpression and micro-vessel formation. Withaferin A's anticancer activity against hepatocellular carcinoma was investigated in athymic nude mouse xenografts. Then, 5 × 106 HepG2 cells were injected subcutaneously into the mice. After 15 days of implantation, mice were divided into the untreated and treatment groups. The untreated group received no treatment, while the treatment group received 4 mg per kg withaferin A orally daily. After 5 weeks of treatment, mice were sacrificed and the tumor was collected and evaluated. Withaferin A showed its anticancer potential by inhibiting Ki67 expression while increasing the ERK, RSK, ELK1, and DR5 levels.^154^ Some recent pre-clinical data related to the anti-cancer potential of plant bio-actives are also listed in Table 4.

**Table 4:** *In vivo* studies of some secondary metabolites from natural sources in different types of cancers

Isolated compound	Plant	Type of cancer	Model/dose	Reference
6-Shogaol	*Zingiber officinale* (Roscoe)	Non-small cell lung cancer	Nude mice model (10 mg kg^−1^)	155
Allicin	*Allium sativum* (Amaryllidaceae)	Liver bile duct carcinoma	BALB/c nude mice model (10 mg kg^−1^)	156
Alpinum	*Derris eriocarpa* (Leguminosae)	Renal cell carcinoma	In BALB/c nude mice xenograft (40 mg kg^−1^)	157
Andrographolide	*Andrographis paniculata* (Acanthaceae)	Breast cancer	Nude (BALB/c females, 6–8 weeks old) mice (25, 50, and 100 mg kg^−1^)	158
Baicalin	*Scutellaria baicalensis* (Lamiaceae)	Colon cancer	Nude mice (50 mg kg^−1^)	159
Curcumin	*Curcuma longa* (Zingiberaceae)	Melanoma cancer	Six-week-old female BALB/c nude mice (25 mg kg^−1^)	160
Decursin	*Angelica gigas* (Apiaceae)	Prostate cancer	SCID-NSG mice xenograft (4.5 mg kg^−1^)	161
Dicumarol	*Melilotus officinalis* (Fabaceae)	Ovarian carcinoma	BALB/c nude mouse xenograft model, DIC (30 mg kg^−1^)	162
Genistein	*Glycine max* (legumes)	Leukemia	Male athymic BALB/c nu/nu mice 6–8 week (0.2 or 0.4 mg kg^−1^)	163
Gingerol	*Zingiber officinale* (Roscoe)	Breast cancer	Mice model (5 mg kg^−1^)	164
Glycyrrhizin	*Glycyrrhiza glabra* (Fabaceae)	Non-small cell lung cancer	Athymic BALB/c nude mice xenograft (100 mg kg^−1^)	165
Hispidulin	*Salvia involucrata* (Lamiaceae)	Hepatocellular carcinoma	Nude mice (20 mg kg^−1^)	166
Stilbenoid	*Polygonum cuspidatum* (Polygonaceae)	Breast cancer	Nude mouse mode (5 mg kg^−1^)	167
Licochalcone A	*Glycyrrhiza glabra* (Fabaceae)	Glioma cell	Athymic nude mice (10 mg kg^−1^)	168
Nimbolide	*Azadirachta indica* (Meliaceae)	Pancreatic cancer	Athymic nu/nu mouse model, (5 mg kg^−1^)	169
Physapubescin B	*Physalis pubescens* L. (Solanaceae)	Renal cell carcinoma	Xenograft mouse model (30 mg kg^−1^)	170
Pterostilbene	*Polygonum cuspidatum* (Polygonaceae)	Endometrial cancer	Xenograft mouse model (30 mg kg^−1^)	171

### 3.4 Clinical trial data for plant-derived bioactives in cancer management

Despite the fact that an enormous number of anti-cancer molecules are currently being developed, clinical trials using phyto-chemicals to manage different cancers are still in the early stages.^172^ The trials on anticancer moieties are focused on three important components: first, increasing cancer cells response to standard chemo- and radio-therapy; second, minimising the severe side effects of traditional cancer therapy; and third, identifying undesirable interactions with standard therapy. Preclinical studies of various phytoconstituents have revealed a high potential for treating various types of cancers. Due to a lack of research and knowledge regarding their mechanism of action, the specific site of action, and dose, they failed to enter clinical trials. Currently, only seven phytoconstituents are under clinical trial as reported in Table 5.^173–175^

**Table 5:** List of various bio-actives derived from plants under various stages of clinical trials for the management of different types of cancers

Isolated compound	Biological source	Type of cancer	Stage of trial	Identifier code
Berberine hydrochloride (alkaloid)	*Berberis* sp. (Berberidaceae)	Colorectal cancer	Placebo-controlled phase 2/3 trial berberine hydrochloride (1000 patients) (300 mg twice per day)	NCT03281096
Curcumin (polyphenol)	*Curcuma longa* (Zingiberaceae)	Advanced and metastatic breast cancer	Placebo-controlled phase 2/3 trial curcumin (300 mg i.v. per day) along with paclitaxel (80 mg per m^2^ BS; i.v.) once a week for 12 weeks	NCT03072992
Epigallocatechin (flavonoids)	*Camellia sinensis* (Theaceae)	Colorectal cancer	Early phase 1 trial Teavigo™ (highly purified and refined green tea extract providing 94% EGCG) (450 mg PO per day)	NCT02891538
Lycopene (carotenoids)	*Solanum lycopersicum* (Solanaceae)	Metastatic colorectal cancer	Placebo-controlled phase 2 trial lycopene (20 mg PO per day) to reduce skin toxicity	NCT03167268
Quercetin (carotenoids)	*Glycyrrhiza glabra* (Leguminosae)	Prostate cancer	Phase 1 trial, placebo-controlled, two arm study of quercetin and green tea to enhance the bioavailability of green tea polyphenols in men scheduled for prostatectomy	NCT01912820
Resveratrol (stilbenoid) (polyphenol)	*Polygonum cuspidatum* (Polygonaceae)	Low-grade GI neuroendocrine tumors	Placebo-controlled phase 1 trail, resveratrol (2.5 g p.o. twice per day) on Notch-1 signaling in low-grade gastrointestinal neuroendocrine tumors	NCT01476592
Sulforaphane (isothiocyanate)	*Brassica oleracea* (Brassicaceae)	Former smokers with a high risk of developing lung cancer	Placebo-controlled phase 2 trial Avmacol (sulforaphane) tablets (120 μM p.o. twice per day)	NCT03232138

### 3.5 Structure–activity relationship (SAR) analysis of compounds with similar structures

Berberine and its derivatives have efficient cytotoxic potential against breast cancer, liver cancer and pancreatic cancer, and its structural analysis shows that its antitumor activity is mainly concentrated on C-9 and C-13, the derivatives being more potent than the parent compound. Propyl benzene and 4,4-diphenylbutyl on C-9 showed better anti-breast cancer cell toxicity, while 1-chloro-4-ethylbenzene and phenylpentyl on C-13 are strong electron-withdrawing groups and strengthen cytotoxicity potential against hepatic cancer cell toxicity compared to the parent compound.^18^

#### Evodiamine and its derivatives

Substitution at different positions of evodiamine such as fluorine at the C3 position, chlorine at both the C3 and C10 positions strengthen the cytotoxity potential. The free hydroxyl group at the 10 position is also important for high antiproliferative activity. Substitutions at C3 and C10 have also been found to have synergistic effects. For example, 3-fluoro-10-hydroxyevodiamine and 3-amino-10-hydroxyevodiamine not only exhibit excellent antitumor activity but also have good water solubility. In addition, modifications of the D-shaped ring framework are also tolerated. Substitution of the C5 carbonyl group with a thio-carbonyl group or substitution of the N14 methyl group with an oxygen atom has a positive effect on antitumor activity.^20^

SAR analysis shows that analogs of pancratistatin depend largely on the hydroxyl group at C-7 and the functional group substitution at C-1. Three new synthetic analogs, SVTH-5, SVTH-6, and SVTH-7, were examined, which possesses the complete anticancer pharmacophore of pancratistatin, including the hydroxyl group at C-7. As a result, SVTH-6 and SVTH-5 were more effective against cancer cells than related compounds JCTH-4 and JCTH 3, respectively, which lack this functional group. In addition, the functional group at C-1 significantly determines the effectiveness of the analogs. For example, JCTH-1 and JCTH-2 differ from JCTH-4 only in the functional group at C-1 and have hardly any anticancer activity. Similarly, SVTH-7 differs from SVTH-6 and SVTH-5 only in the C-1 group and is more effective against most cancer cell lines tested.^43^

#### Antiaroside and its derivatives

The structure–activity relationships of these compounds showed that the orientation of the C-3 and C-17 substituents plays an important role in the overall cytotoxicity profile. However, compounds with α-orientation of the C-3 and C-17 substituents showed weaker anticancer activity. Similarly, compounds containing an α-l-rhamnose residue at C-3 showed potent cytotoxic activity. The position of the glycosyl bond is also very important for cytotoxicity. For example, compounds with the sugar attached to C-19 showed lower cytotoxicity than compounds with this residue attached to C-3.^76^

Cymarin, hyrcanoside and deglucohyrcanoside contain a carbonyl group at C-19 and a β-hydroxyl group at C-5. While ouabain contains a β-hydroxyl group at C-1, α-OH groups at C-10 and C-19, but lacks a β-OH group at C-5. The β-hydroxyl group at C-5 may contribute to general cytotoxicity. However, as reported in the literature, cytotoxicity is significantly affected by the carbonyl group at C-19; when the hydroxyl group at C-19 is replaced by a carbonyl group, the cytotoxicity of the resulting derivative increases.^80^

#### Sophoridine and its derivatives

From the SAR studies, it can be concluded that the substitution of a phenylmethylene group at the C-14 position of the parent sophoroside resulted in enhanced anticancer activity. The addition of a conjugated structure at the C-15 position of the carbonyl group *via* imine formation resulted in enhanced anticancer activity. The substitution of fatty acyl group at the C-12 position significantly improved the antitumor activity of *N*-substituted sophoroside derivatives. However, the side chain at the C-12 position is larger and not suitable for anticancer activity.^176^

## 4 Discussion

**Fig. 7:**
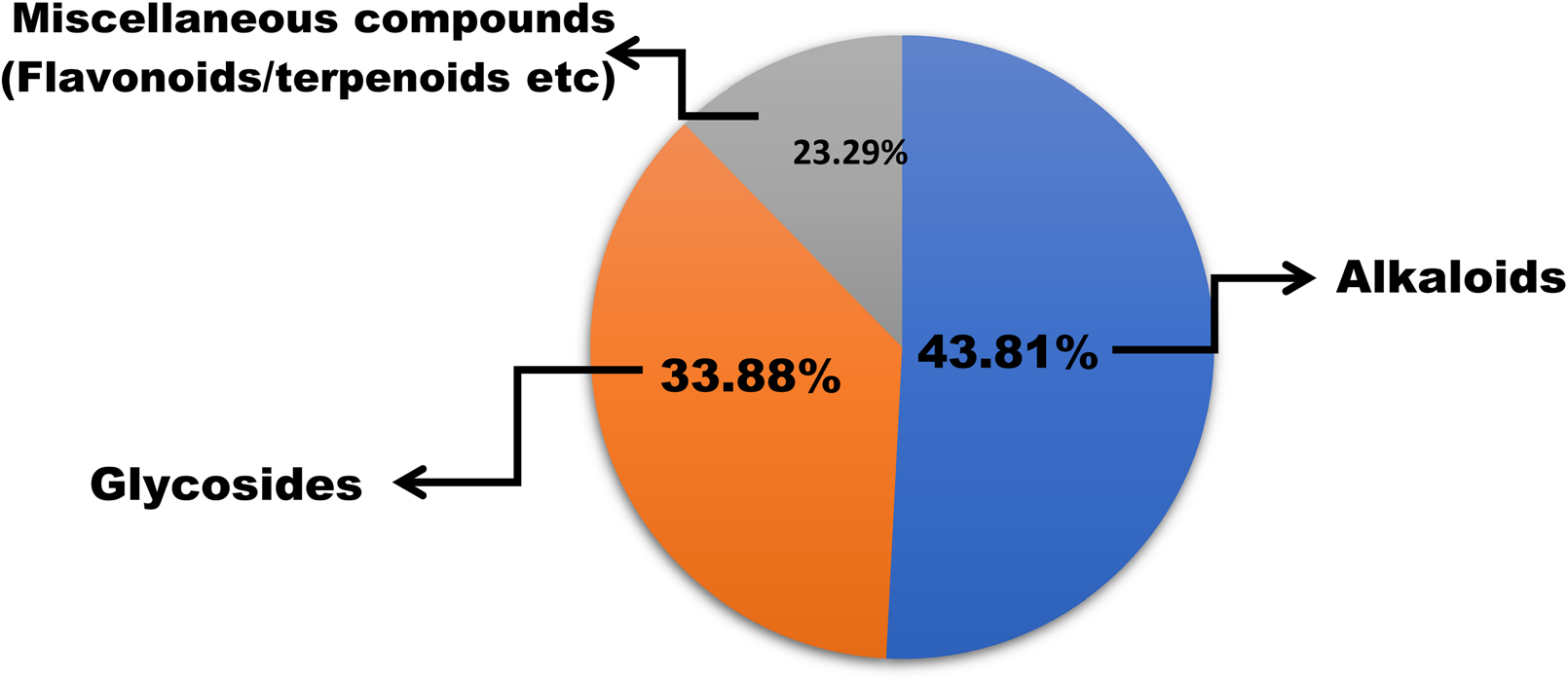
Percentage of different categories of bioactive compounds with anticancer potential.

In this work, we reviewed a total of 144 isolated compounds which are having anticancer potential against different cell lines. Out of 144, there are 62 alkaloids, 47 glycosides, and 35 other isolated compounds, which include flavones, terpenoids, terpene, and polyphenols (Fig. 7). Out of 62 alkaloids, only one alkaloid berberine is currently under clinical trials in placebo-controlled phase 2/3 on colorectal cancer.

The IC_50_ value of berberine is 250 nM for breast cancer cell lines, 400 nM for colorectal cancer, and 200 nM for chondrosarcoma and lung cancer. Berberine's structural alteration for anticancer action had primarily focused on C_9_ and C_13_. To increase the efficacy and bioavailability, some cycloberberine derivatives were also developed. By enhancing the moderate DNA-binding affinities of protoberberine alkaloids, five derivatives substantially inhibit human HepG2 and human colon cancer cell lines. With an IC_50_ of 200 nM, evodiamine has anticancer potential in human liver cancer cell lines (HepG2 and PLHC-1). The best derivative was 4-chlorobenzene, which had IC_50_ values of 8.6, 4.9, and 260 nM against A549 (lung cancer), MDA-MB435 (breast cancer), and HCT116 (colon cancer) cell lines with *N*-substitution series of evodiamine derivatives. Bulbispermine shows cytotoxic effects against glioblastoma (T98G and U373) and human leukemia (HL-60) with IC_50_ values of 90 nM, 380 nM, and 80 nM. Distichamine shows anticancer activity against HeLa, CEM, K562, MCF-7, and G-361 with IC_50_ values of 22–147 nM. Lycorine had IC_50_ ranging from 50 nM to 100 nM for different cell lines such as PC-3M, DU145, LNCaP, and 22RV1 and *in vivo* lycorine (5 mg per kg per day or 10 mg per kg per day) reduces prostate cancer.

We have also reviewed 47 glycosides that have anticancer potential against various cancer cell lines and digoxin was found to have the greatest potential to treat various cancers. Digoxin has undergone 27 clinical trials in which 11 trails are completed, 7 trails are under recruiting, 1 trail is not yet recruiting, 2 trails are active, 2 trails are terminated, 3 studies have unknown status, and 1 study is withdrawn. All trials involved digoxin alone or a combination of digoxin with other drugs such as enzalutamide, rosuvastatin, capecitabine, lapatinib, metformin, and simvastatin. These trials are conducted on various cancer cell lines such as prostate, head and neck, pancreatic lung, and breast cancer on neoplasm and solid tumours. Ouabain had IC_50_ values for H460 and PANC1 of 10.44 nM and 42.36 nM respectively. The IC_50_ value of bufalin is 20.0 nM for breast cancer, 16.6 nM for cervical cancer, 28.23 nM for gallbladder cancer and 15.57 nM for lung cancer. Imperatorin inhibits colon cancer with an IC_50_ value of 78 nM. In combination with quercetin, imperatorin showed the synergistic effect by reducing the cell viability of HeLa cells to 52.86% and for Hep-2 cells to 39.34%. Esculetin inhibits the HCC cell with an IC_50_ value of 2.24 nM and reduces the tumor growth by 20.33, 40.37, and 55.42% in Hepa1-6 cell-containing mice. Cardiac glycosides such as digitoxin, digitoxigenin, lanatoside, oleandrin and neritaloside showed anticancer potential with significant IC_50_ values of 22 nM, 15 nM, 19 nM, 50 nM, and 90 nM on lung cancer (A549 and H1975), and of 59 nM, 43 nM, 45 nM, 1104 nM, and 165 nM on osteosarcoma (U2OS and SaOS-2) respectively.

Amongst miscellaneous isolated compounds epigallocatechin curcumin, lycopene, and resveratrol are under clinical trials. Totally 32 clinical trials were studied on epigallocatechin, of which 16 studies are completed, 6 trials are under recruiting, 1 study is enrolled by invitation, 1 study is active but this study is not recruiting, 6 studies are terminated and 2 trials are withdrawn. Camphor has one completed clinical trial related to the feasibility of recruiting pediatric patients receiving chemotherapy for cancer towards homeopathy. Fisetin has one clinical trial and this trial is not yet recruiting, which is related to the efficacy of the combination of dasatinib with quercetin and fisetin to reduce senescence and to improve frailty in adult survivors of childhood cancer. Silymarin has 6 clinical trials and all 6 are completed, and these trials were for colorectal, breast cancer, prostate cancer, upper GI cancer, colon, and leukemia. A total of 17 clinical trials have been done on combretastatin, in which 11 studies are completed, 4 studies are terminated, 1 trail has unknown status and 1 trail is withdrawn. These trails include head and neck cancer, sarcoma, neuroendocrine tumor, and solid tumors. Linalool shows anticancer potential against prostate cancer with IC_50_ values of 28.3 and 10.5 nM at 24 h and 48 h respectively.

**Fig. 8:**
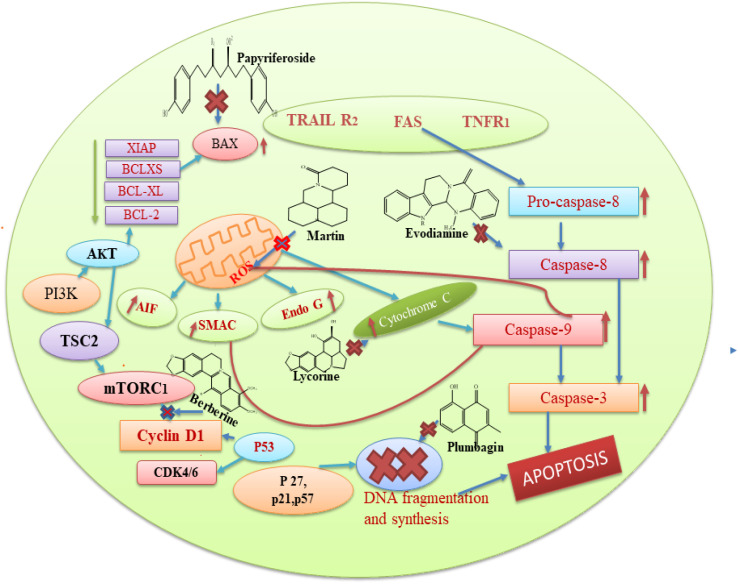
Basic mechanism of action of some bioactive compounds in cancer management.

Based on the aforementioned findings from the literature, we can state that each plant bioactive has a different method of action that targets different cancer cells. Alkaloids typically prevent cancer by blocking the replication of DNA and causing protein denaturation, and this leads to apoptosis. Moreover, alkaloids inhibit the caspase inhibitor and the G_2_/M phase, while glycosides decrease the proliferation of cells by altering the expression of IB phosphorylation, BCL-2, caspase 3, and BAX proteins. Cardiac glycosides block the transport of sodium ions across the membrane, and this causes an increase in the concentration of calcium ions in the plasma membrane, which is involved in the regulation of multiple signal pathways, including apoptosis. The basic mechanism of action of these secondary metabolites is also represented in Fig. 8. Among the seven compounds currently undergoing clinical trials, curcumin, resveratrol and berberine stand out as the most significant. Clinical investigations of curcumin are assessing its efficacy against various cancers, such as colorectal, pancreatic, and breast cancers. However, curcumin's low solubility in water restricts its absorption within the gastrointestinal tract, leading to a low concentration of the compound in the bloodstream and posing challenge in achieving therapeutic levels.^177^ Researchers are actively seeking methods to improve its bioavailability, given that the natural form of curcumin is poorly absorbed by the body. Additionally, there is some evidence regarding the ideal dosage and administration frequency for curcumin, especially in the context of cancer treatment. This ambiguity complicates the design of clinical trials and hinders the ability to compare findings across different studies. In the case of resveratrol, clinical trials are investigating its potential in the prevention or treatment of cancers, including breast, prostate, and colon cancer. However, similar to curcumin, the low bioavailability of resveratrol presents a challenge for therapeutic use, with resveratrol being rapidly metabolized and excreted from the body, resulting in low plasma concentrations. It is extensively metabolized in the liver and intestines, limiting the amount that enters the bloodstream and target tissues.^178^ While resveratrol is generally considered safe at lower doses, higher doses may cause side effects, such as gastrointestinal upset and, in some cases, kidney damage. Clinical investigations of berberine are currently underway to test the effectiveness of berberine in various types of cancer, including lung and colon cancer, as well as to determine its potential synergistic effects with conventional chemotherapy. Berberine can inhibit enzymes involved in drug metabolism, such as cytochrome P450 enzymes, which may interact with other medications and reduce their effectiveness or increase the risk of side effects.^179^

## 5 Conclusion

Medicinal plants contain a significant number of secondary metabolites belonging to various categories such as alkaloids, glycosides, flavonoids, terpenes, and terpenoids. These compounds have shown promising anticancer properties against multiple types of cancers. Extensive literature reviews have indicated that each active compound from plants exhibits a distinct mechanism of action in the treatment of different cancers. Additionally, certain phytoconstituents such as vinca alkaloids, taxane diterpenoids, camptothecin derivatives, and epipodophyllotoxin are currently utilized in cancer therapy. Meanwhile, berberine, curcumin, lycopene, quercetin, resveratrol, and sulforaphane are currently under clinical trials. In this work, the authors reviewed a large number of secondary metabolites, which play an important role in preventing and treating various types of cancers and are under different stages of clinical trials. To conclude, we can say that, plant-derived bioactives hold tremendous anticancer potential, which could lead to the establishment of novel therapeutic agents. However, persistent study is required to discover the uncovered moieties of new plants with anticancer potential, which may offer breakthrough for improving anticancer therapy.

## Data availability

No primary research results have been included and no new data were generated or analysed as part of this review.

## Conflicts of interest

All the authors declared no conflict for the submitted manuscript.
